# Supplementary material for: Profiling temporal dynamics of acetogenic communities in anaerobic digesters using next-generation sequencing and T-RFLP
Source: Sci Rep. 2021 Jun 24;11:13298. doi: 10.1038/s41598-021-92658-2 (PMC8225771; doi:10.1038/s41598-021-92658-2)
Supplement: Supplementary file 2 — Supplementary Information 2. [file 41598_2021_92658_MOESM2_ESM.docx]

# Supplementary Figures

**Profiling temporal dynamics of acetogenic communities in anaerobic digesters using next-generation sequencing and T-RFLP**

Abhijeet Singh^1*^, Bettina Müller^1^, Anna Schnürer^1*^

^1^Anaerobic Microbiology and Biotechnology Group, Department of Molecular Sciences, Swedish University of Agricultural Sciences, Almas Allé 5, Uppsala, SE-750 07, Uppsala, Sweden

*For correspondence. E-mail: abhijeet.singh@slu.se; anna.schnurer@slu.se; Tel. +46 18671000; Fax +46 18672000; Address: Department of Molecular Sciences, Box 7025, 75007 Uppsala, Sweden

**A)**


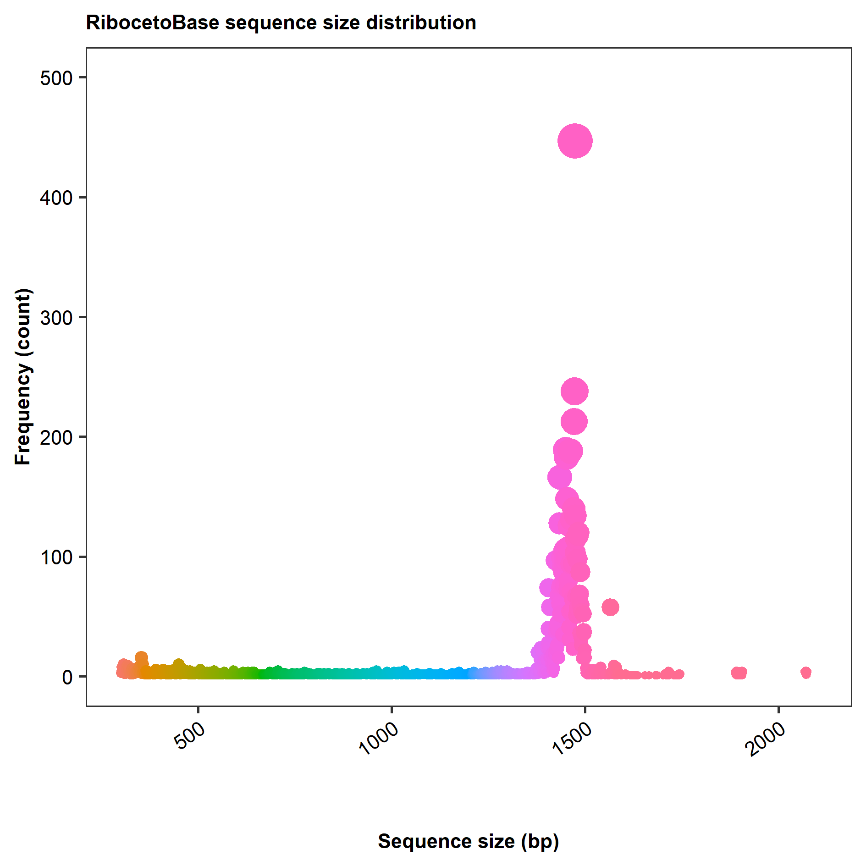


**B)**


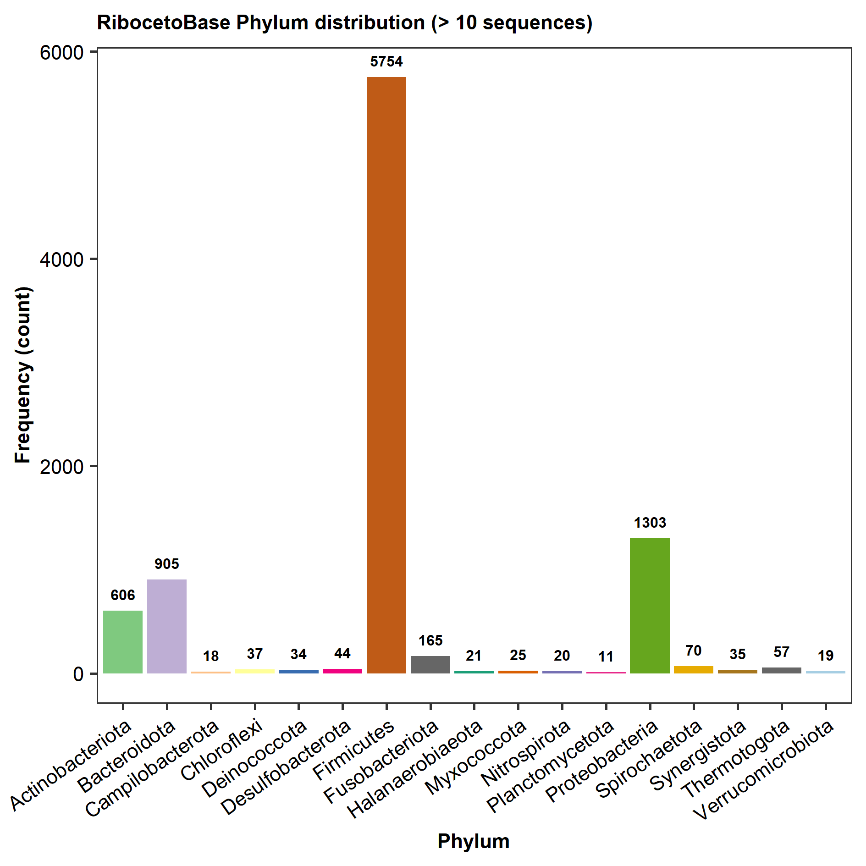


**Supp. Figure S1** - Plots describing the distribution of 16S rRNA sequences present in RibocetoBase according to **A)** lengths **B)** associated taxonomy


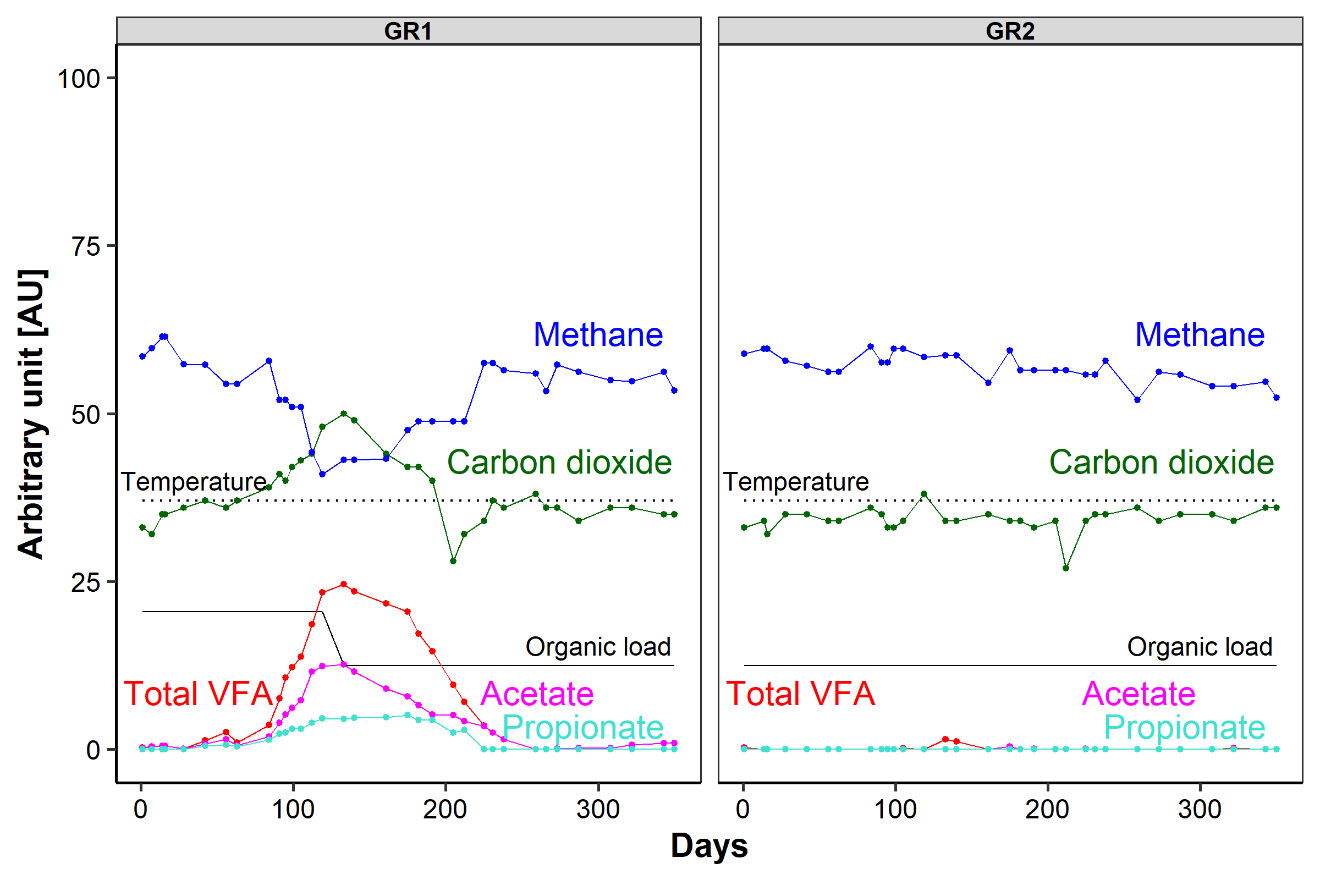


**Supp. Figure S2** - Line chart representing reactor performance of the experimental (GR1) and control (GR2) reactor showing the content of methane (%) and carbon dioxide (%) and concentration of total volatile fatty acid (VFA), acetate and propionate concentration (g/L). The black solid line represents the organic load (20.47 & 12.45 g VS/day) and dotted line represent the operating temperature (37 °C) of the reactors.

**A) B**
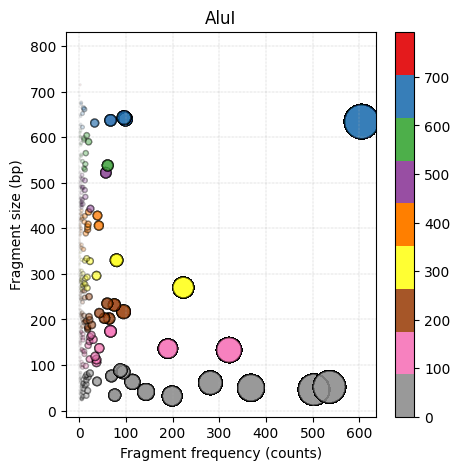

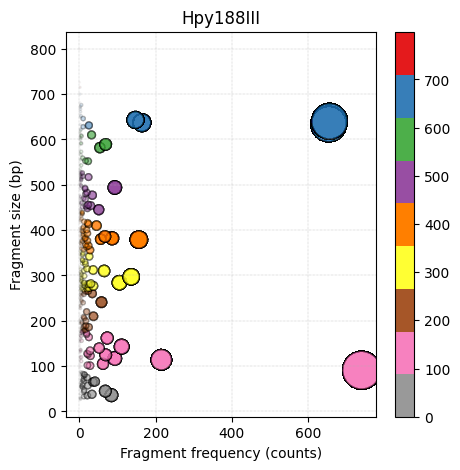


**Supp. Figure S3** - Scatter Plot of *in silico* terminal restriction fragments from AcetoBase formyltetrahydrofolate synthetase (FTHFS) reference dataset generated by the REDigest program with the restriction enzyme **A)** AluI and **B)** Hpy188III.

**A B**


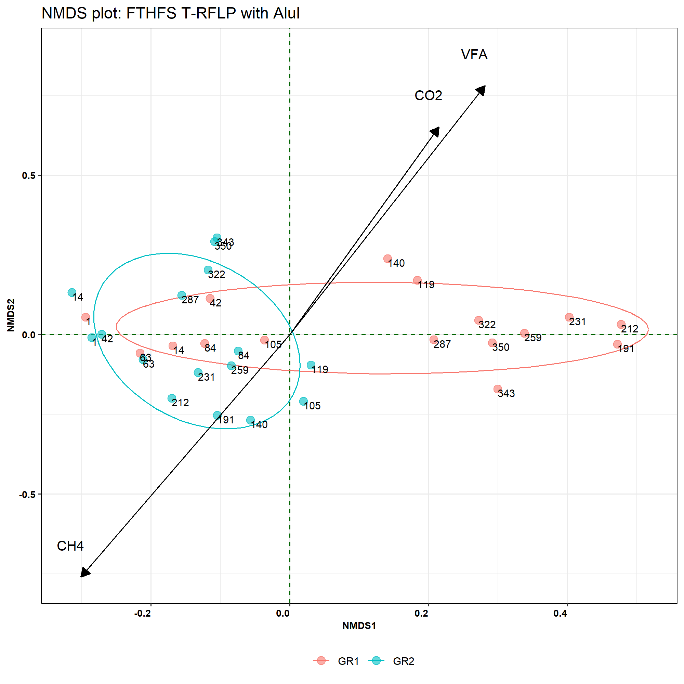

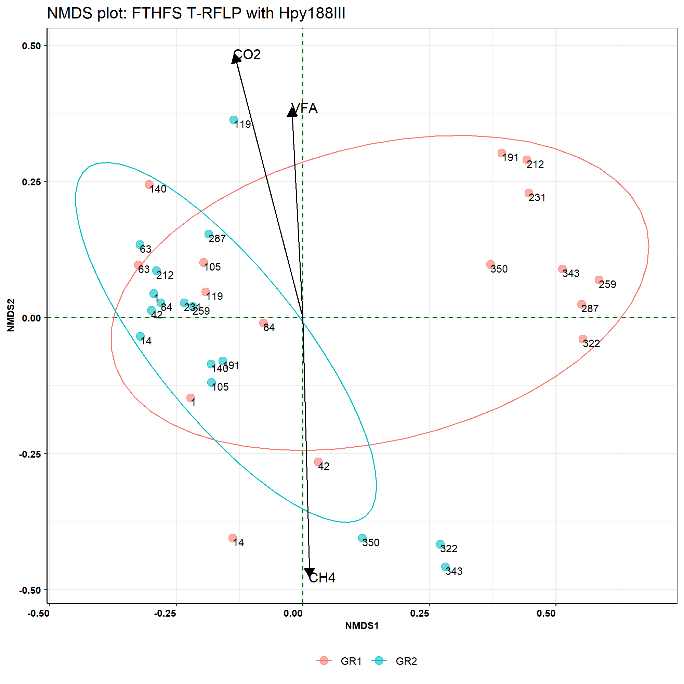


**C D**


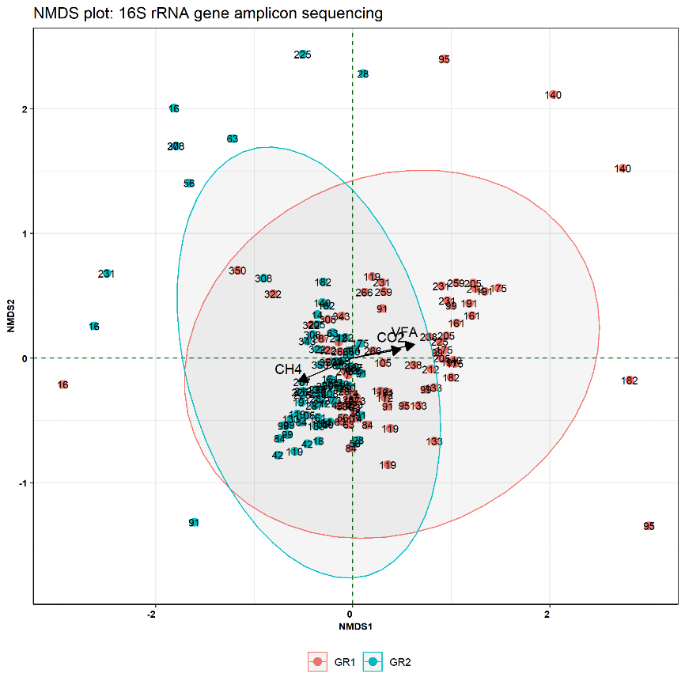

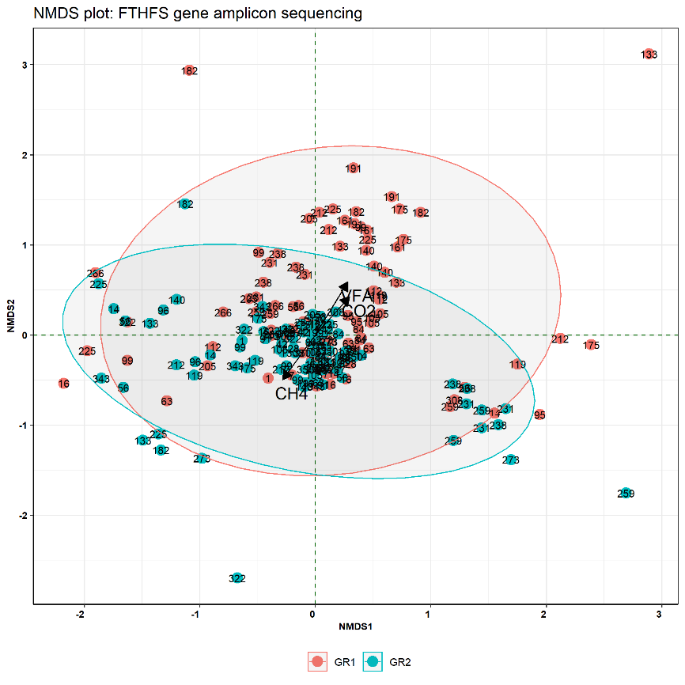


**Supp. Figure S4** - Non-metric Multidimensional Scaling (NMDS) plot representing the microbial beta diversity within the experimental (GR1) and control (GR2) reactors using FTHFS gene terminal restriction fragment length polymorphism (T-RFLP) profile with restriction enzyme **A)** AluI and **B)** Hpy188III. NMDS plot for the Next-generation sequencing of **C)** 16S rRNA gene amplicons and **D)** FTHFS gene amplicons. Numbers in NMDS plot represent the sampling points.

**
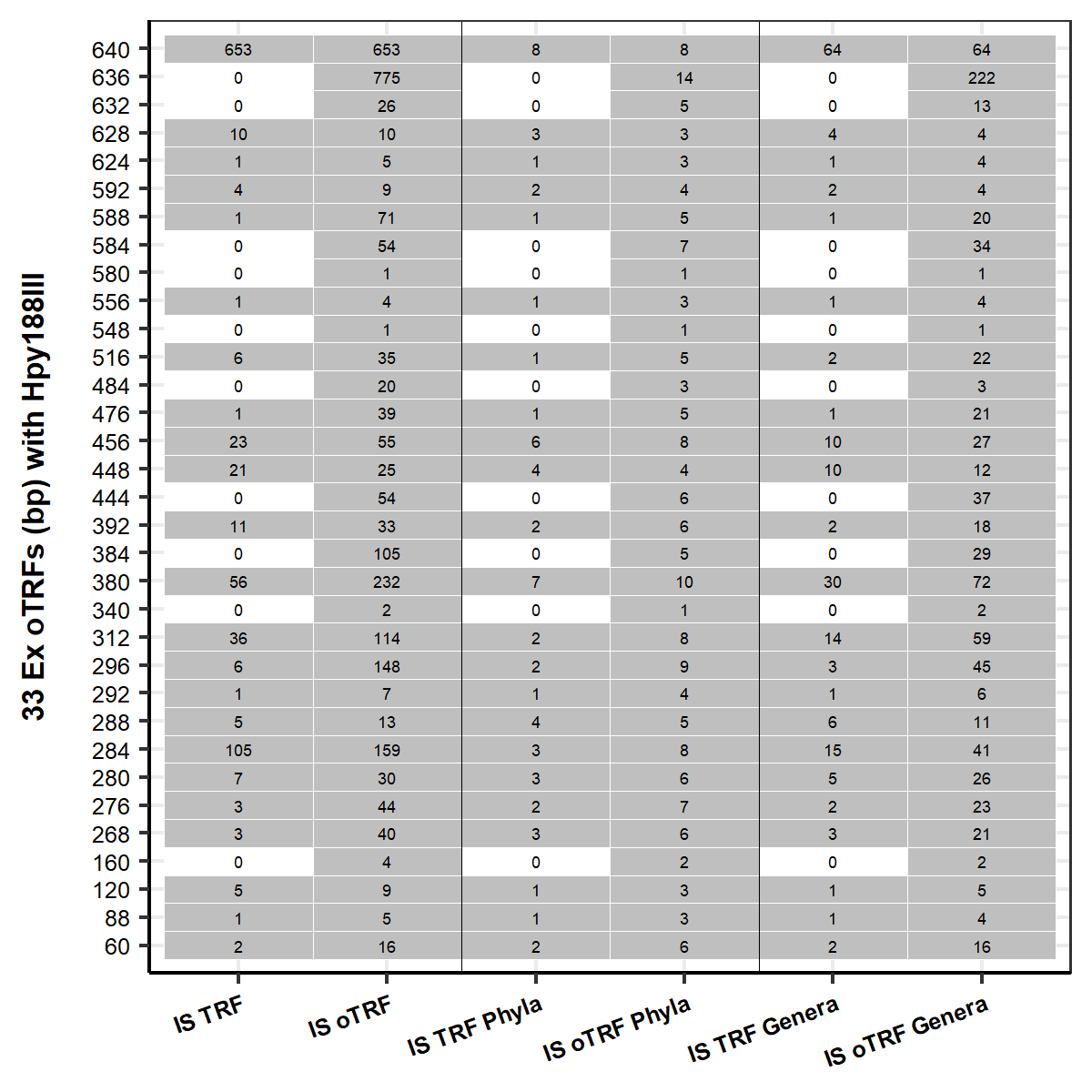
**

**Supp. Figure S5** - Tabular plot representing FTHFS gene T-RFLP profile during comparison of 33 experimental operational terminal restriction fragment (*Ex* oTRF) in base pairs (bp) versus counts of *in silico* (*IS*) TRF, *IS* oTRF and counts of taxa (phyla and genera) with restriction enzyme Hpy188III.

**A) B)**


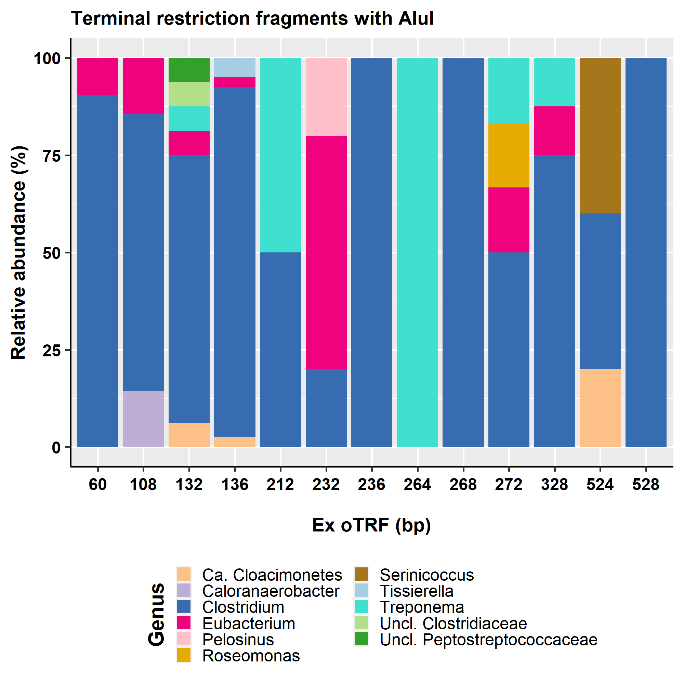

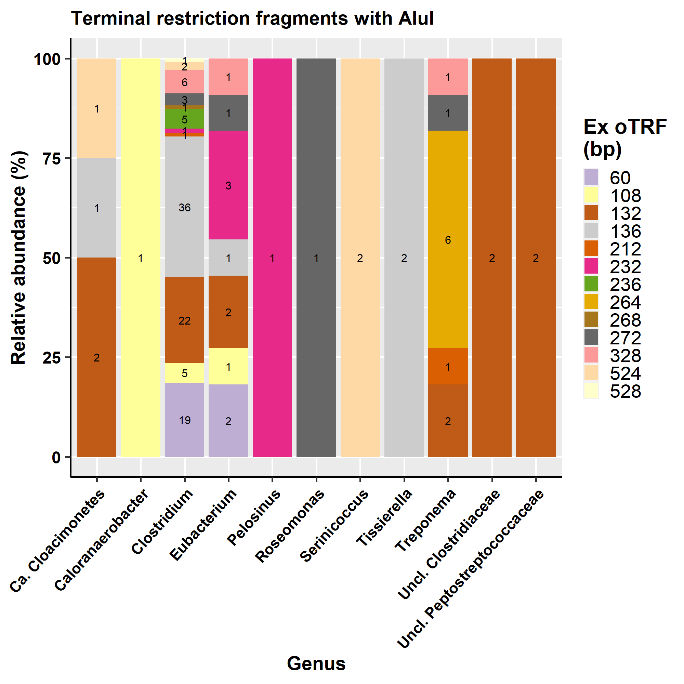


**Supp. Figure S6** - Bar plot representing the taxonomic predictions of the major Experimental operational terminal restriction fragments (*Ex* oTRFs) of FTHFS gene generated with the restriction enzyme AluI. A) show the relative abundance of different genus representing the respective *Ex* oTRFs, B) indicate the count based relative abundance of *Ex* oTRFs represented by respective genus in the T-RFLP profile.


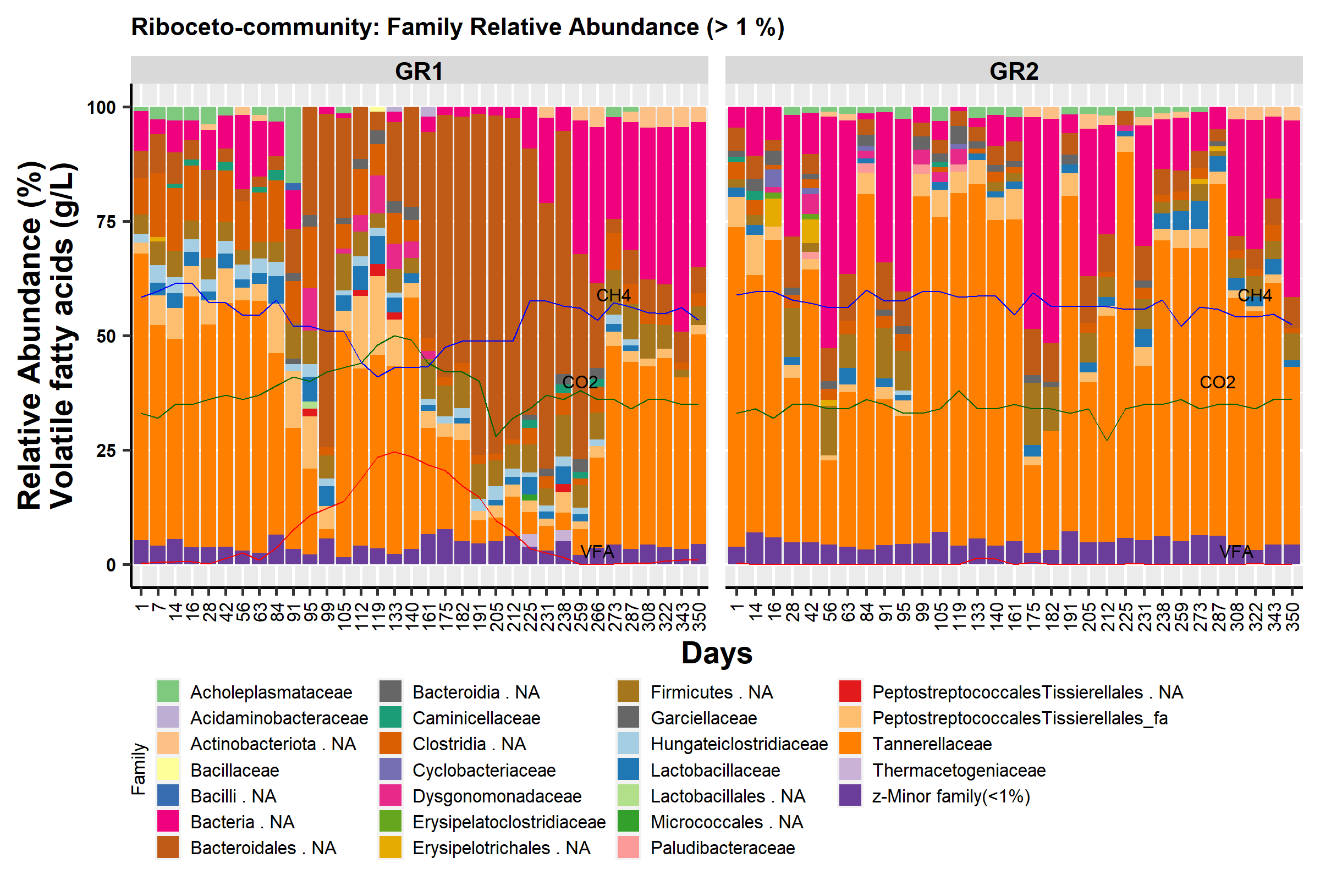


**Supp. Figure S7** - Bar plot representing Riboceto-community in experimental reactor GR1 and control reactor GR2 at the family level (Relative abundance (RA) >1%). VFA, CH_4_ and CO_2_ represent the level of total volatile fatty acids (g/L), methane content (%) and carbon dioxide content (%), respectively.


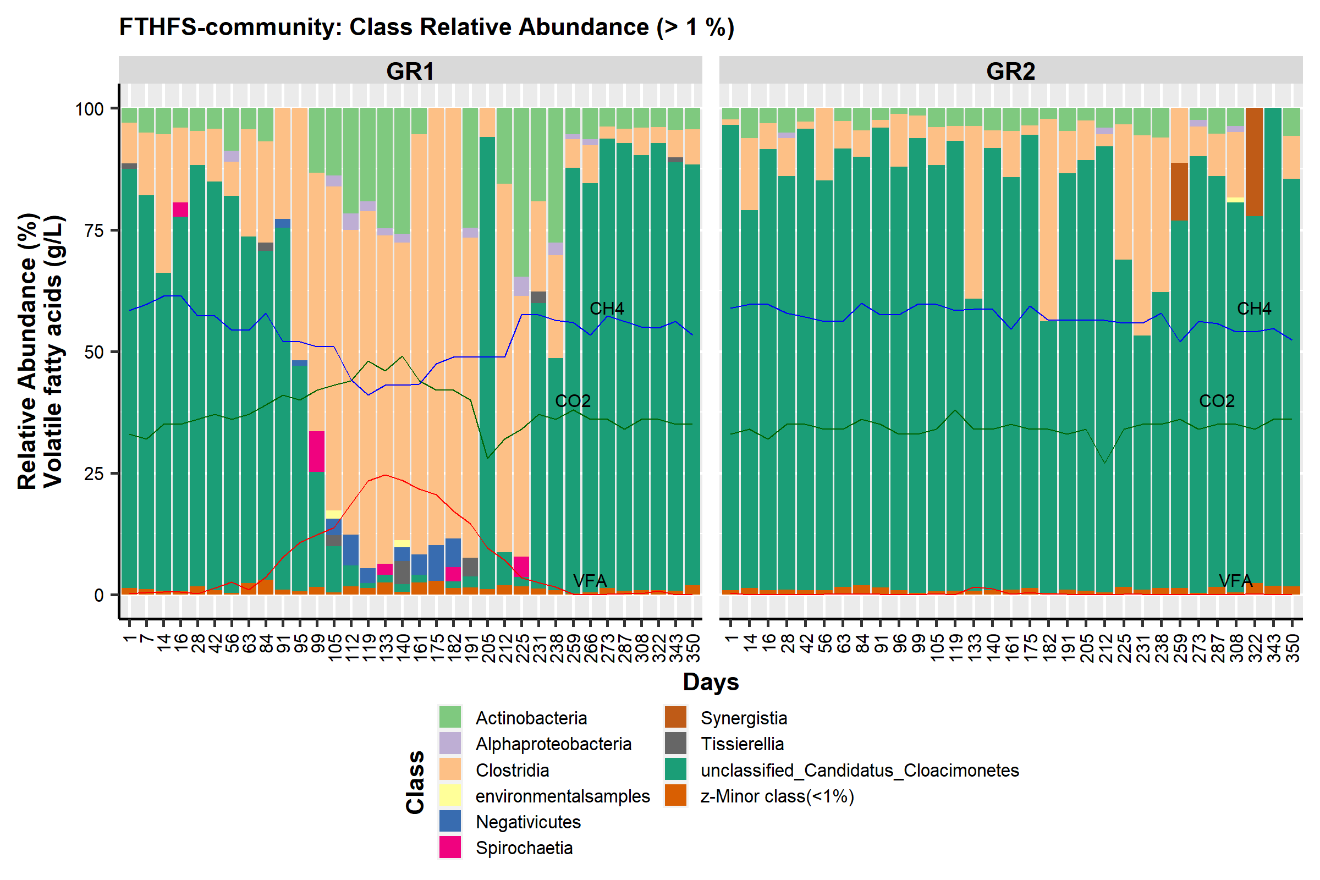


**Supp. Figure S8** - Bar plot representing FTHFS-community in experimental reactor GR1 and control reactor GR2 at the class level (Relative abundance (RA) >1%). VFA, CH_4_ and CO_2_ represent the level of total volatile fatty acids (g/L), methane content (%) and carbon dioxide content (%).


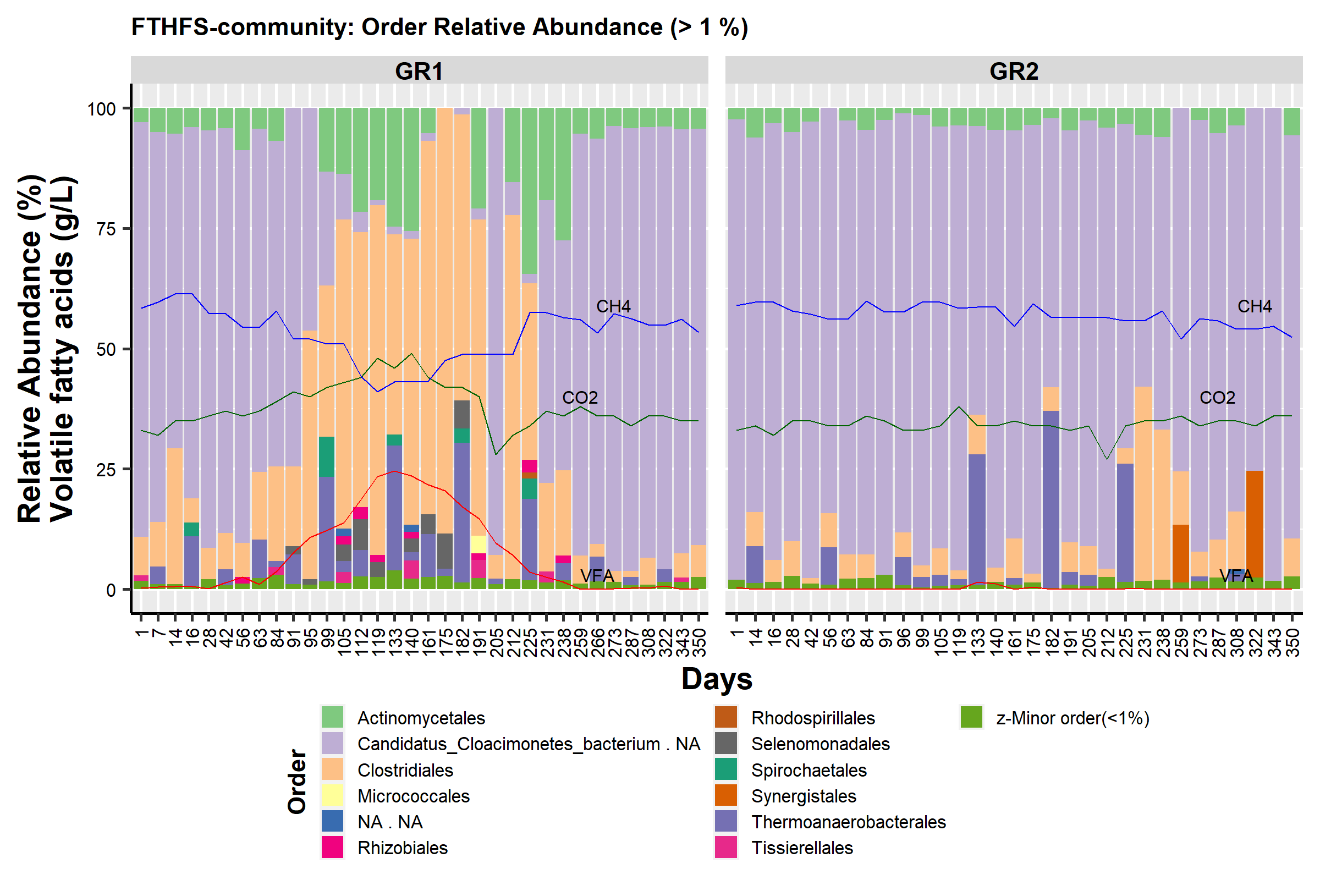


**Supp. Figure S9** - Bar plot representing FTHFS-community in experimental reactor GR1 and control reactor GR2 at the order level (Relative abundance (RA) >1%). VFA, CH_4_ and CO_2_ represent the level of total volatile fatty acids (g/L), methane content (%) and carbon dioxide content (%).


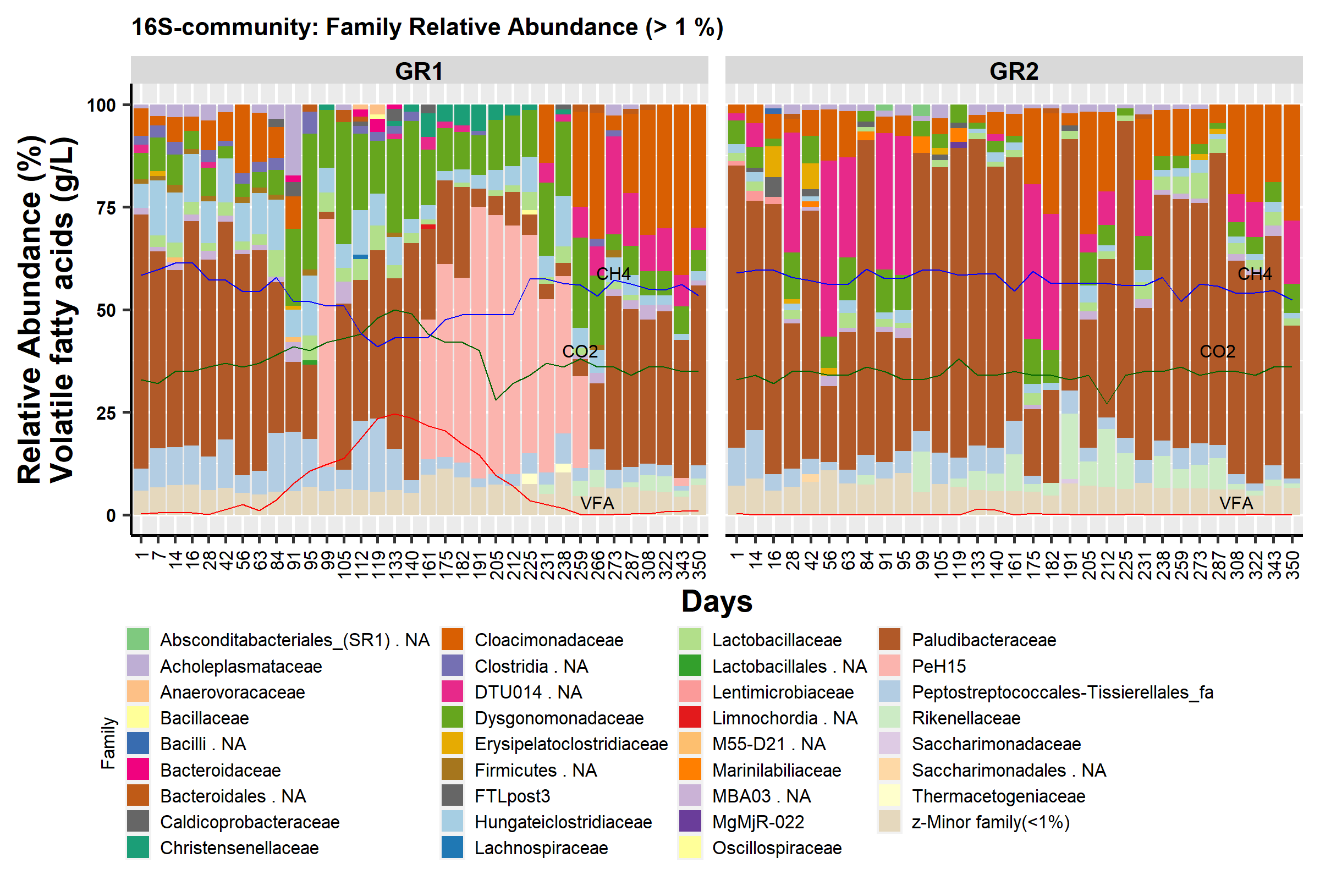


**Supp. Figure S10** - Bar plot representing 16S-community in experimental reactor GR1 and control reactor GR2 at the family level (Relative abundance (RA) >1%). VFA, CH_4_ and CO_2_ represent the level of total volatile fatty acids (g/L), methane content (%) and carbon dioxide content (%).

**A)**

**
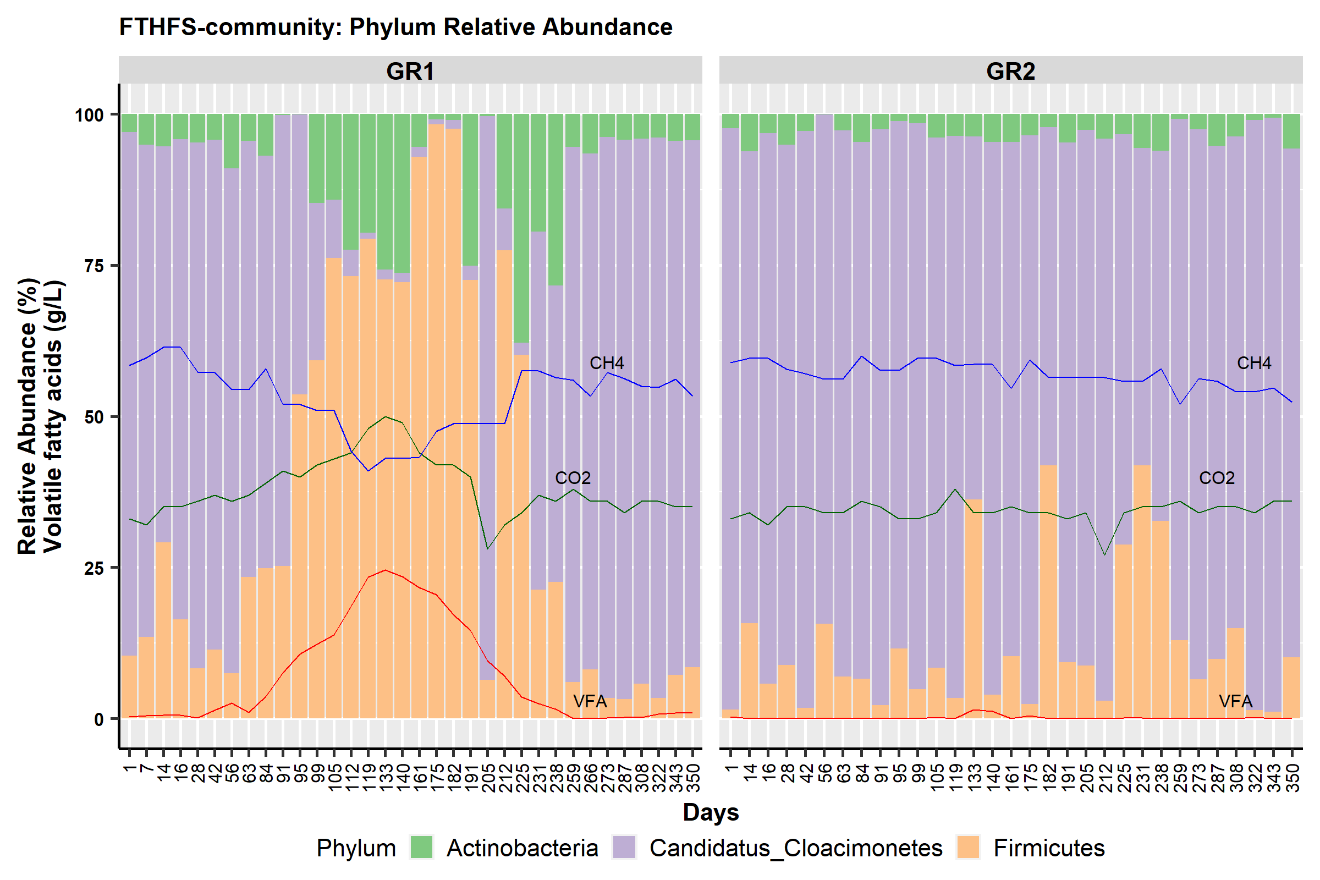
**

**B)**

**
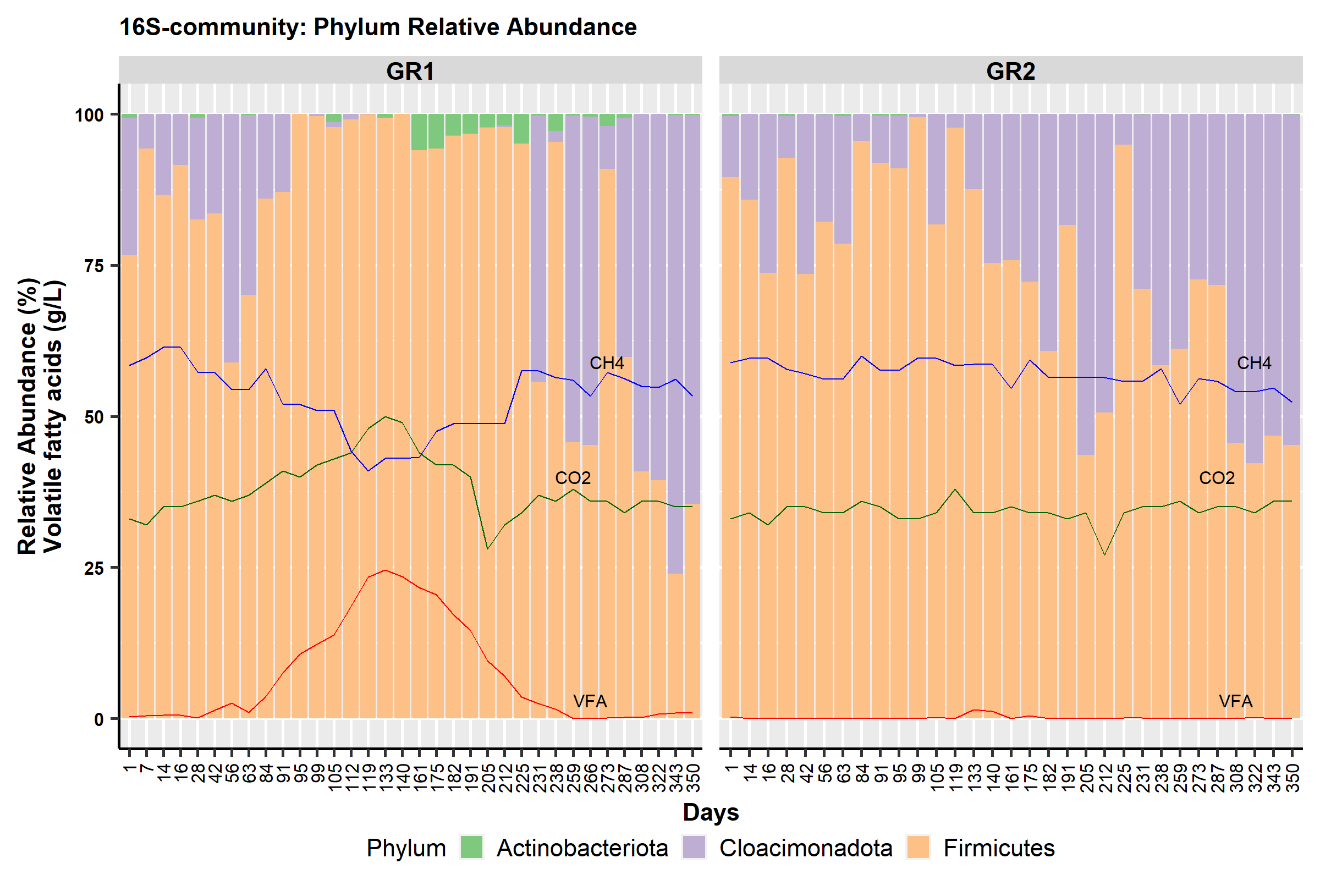
**

**C)**

**
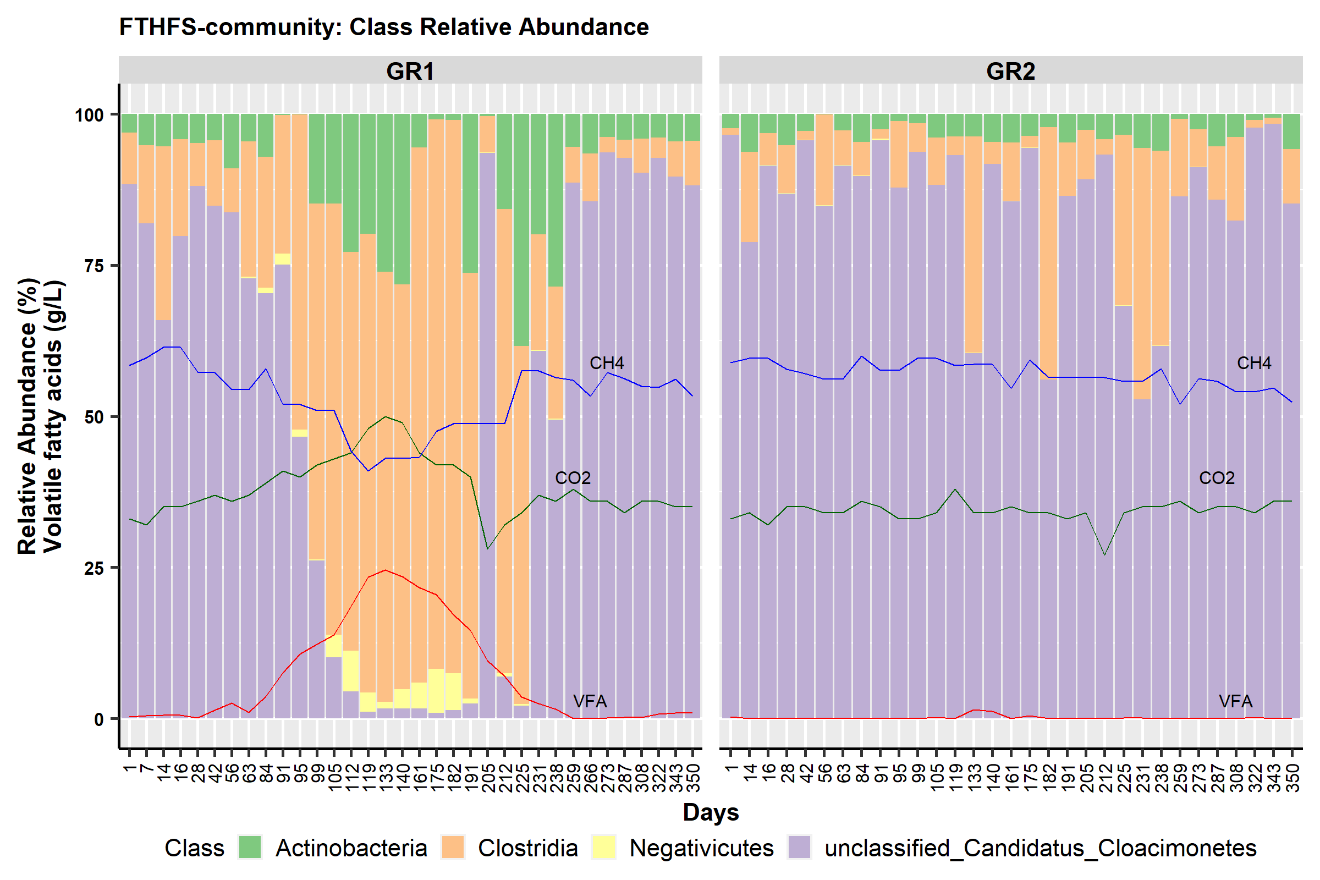
**

**D)**


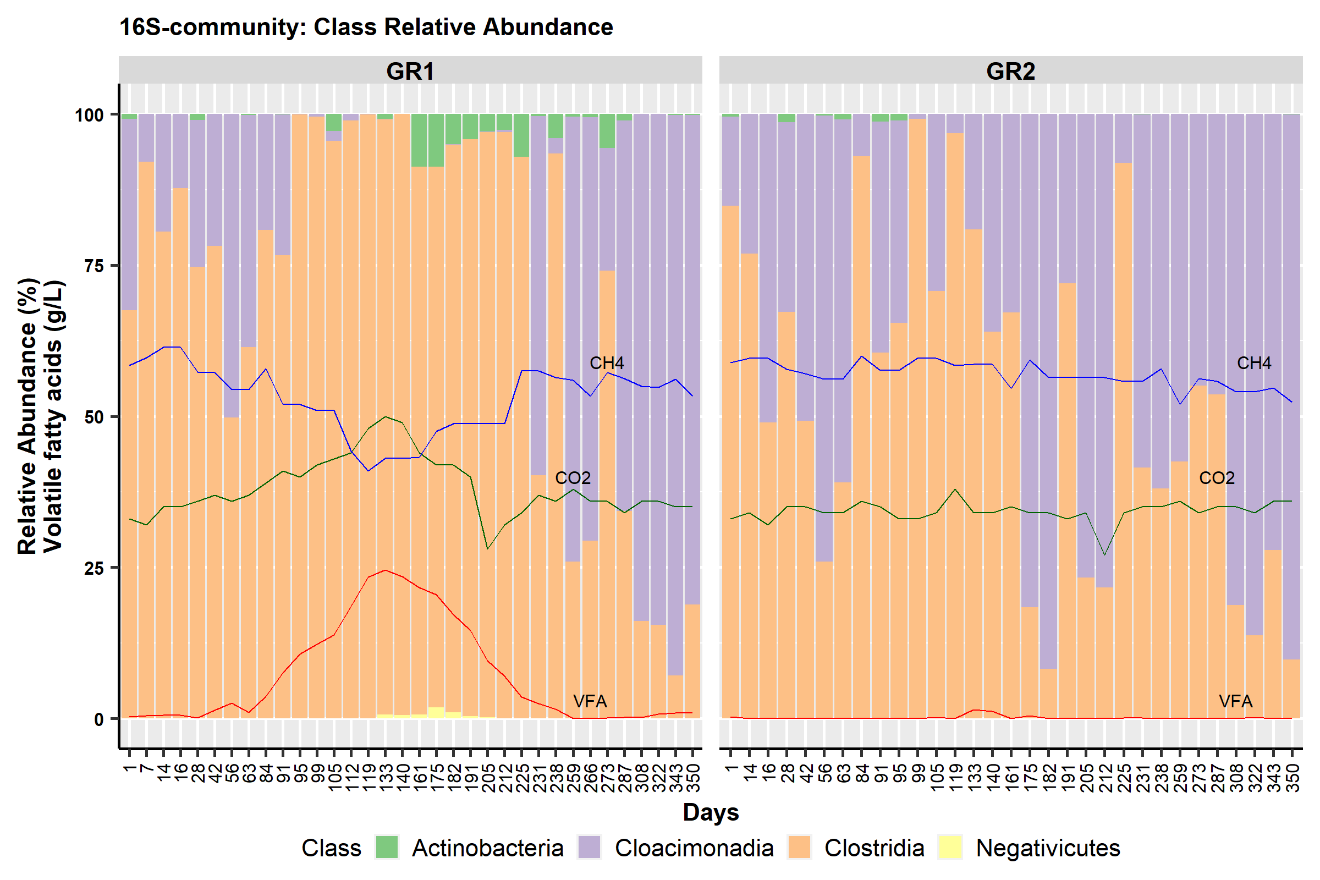


**Supp. Figure S11** - Bar plot representing the top **A)** and **B)** common phyla of FTHFS- and 16S-community, respectively. **C)** and **D)** common classes of FTHFS- and 16S-community, respectively.
